# Association between the dietary inflammatory index and bone markers in postmenopausal women

**DOI:** 10.1371/journal.pone.0265630

**Published:** 2022-03-17

**Authors:** Daeun Song, Jieun Kim, Minji Kang, Jungwon Park, Haelim Lee, Deog-Yoon Kim, So Young Park, Hyunjung Lim

**Affiliations:** 1 Department of Medical Nutrition, Graduate School of East-West Medical Science, Kyung Hee University, Yongin, Republic of Korea; 2 Research Institute of Medical Nutrition, Kyung Hee University, Seoul, Republic of Korea; 3 Department of Nuclear Medicine, Kyung Hee University School of Medicine, Seoul, Republic of Korea; 4 Department of Endocrinology & Metabolism, Kyung Hee University Hospital, Seoul, Republic of Korea; Ehime University Graduate School of Medicine, JAPAN

## Abstract

**Introduction:**

The health problem of postmenopausal women is emerging as an important problem due to the increased aging population. This study investigated the association between dietary inflammatory index (DII) and bone markers in postmenopausal women.

**Methods:**

A cross-sectional study was conducted on 132 postmenopausal women aged 45–70 years. The DII score was calculated using the 3-day food records and divided into tertiles according to the DII score. The lifestyle factors that could affect bone mineral density (BMD) in postmenopausal women were investigated and included the EuroQol- 5 Dimension (EQ-5D), physical activity, and eating habits. Skeletal muscle index-weight (SMIw) was used to evaluate skeletal muscle mass, and alkaline phosphatase (ALP), bone-specific alkaline phosphatase (BSALP), and phosphorus (P) measured as bone biomarkers. The BMD was measured using dual-energy X-ray absorptiometry, and the association between anthropometric, biochemistry, BMD, and DII was assessed.

**Results:**

In the anti-inflammatory group, a high intake of fiber, vitamins, and minerals was observed. After adjusting for confound factors, with higher DII score, percent body fat increased (β = 0.168, p = 0.012), and SMIw decreased linear regression analysis (β = −0.329, p = 0.037, respectively). For biochemistry, confound factors were adjusted, with higher DII score, ALP, BSALP and P decreased and DII score increased (β = −0.057, p = 0.002, β = −0.167, p = 0.004, β = −1.799, p = 0.026, respectively). The relationship between DII and BMD was not significant, but osteopenia increased as DII score increased.

**Conclusion:**

The low DII score is positively associated with low body fat, high muscle mass, elevated bone markers, and low risk of osteopenia.

## Introduction

As life expectancy increases, the aging population is gradually increasing [1]. The health management of middle-aged women is essential because women live one-third of their lifetime in a menopausal state [2]. Women’s bone loss accelerates as the bone resorption rate and increases faster than bone formation with estrogen decline [3, 4]. Osteoporosis is an aging-related bone disease commonly in postmenopausal women [3]. This can lead to the risk of fractures, falls, and complications even with minor trauma.

Osteoporosis can be predicted from bone biomarkers and bone mineral density (BMD) [5]. Bone formation markers are alkaline phosphatase (ALP), osteocalcin (OC), bone-specific alkaline phosphatase (BSALP). And bone resorption marker is C-telopeptide of type 1 collagen (CTx). ALP is an enzyme that plays an important role in bone formation and mineralization [6]. Serum Osteocalcin is considered as a specific marker of osteoblast function, as its levels correlate with bone formation rates [6]. Bone-specific alkaline phosphatase (BSALP) isoenzyme is a clinically preferred marker because of its higher specificity [6]. C-telopeptide of type 1 collagen (CTx) is a sensitive biomarker of bone resorption that can rapidly monitor response to treatment for postmenopausal osteoporosis [7].

Bone mineral density (BMD) can be influenced to decrease by various factors, including nutritional status, as well as genetics, race, age, alcohol intake, smoking status, hormones, and physical activity (PA) [8–11]. Of these, nutritional factors are one of the modifiable factors [4]. Indeed, a sufficient intake of vegetables and fruits is associated with bone health [12–15].

Inflammation can be influenced depending on the food or nutrients consumed [4, 13]. Typically, food that causes inflammation is refined grains, processed meats, high consumption of butter, and high-fat dairy products; conversely, food corresponding to defense inflammation is whole grains, vegetables, fish, and olive oil [13, 16, 17]. Western or meat-based diets are associated with increased inflammation. While Mediterranean-based or vegetable-based diets may reduce the inflammatory markers [18–21]. A pro-inflammatory diet causes low-grade inflammation and elevates inflammatory biomarkers such as C-reactive protein (CRP) and interleukin-6 (IL-6) [21].

A dietary inflammatory index (DII©) is a tool developed based on the actual human consumption intake, focused on the inflammatory properties of these diets [22, 23]. The tool can be used to estimate inflammation levels through dietary intake. It can be used as a tool to measure the probability of inflammation of the diet by applying it to all groups collected through various dietary survey methods. Recently, studies on the relationship between the possibility of inflammation of diet and chronic diseases such as cardiovascular disease, metabolic syndrome, and cancer have been actively conducted using DII [24–27]. A high DII score had a positive association with low BMD and increased risk of fracture [21, 28, 29]. However, the relationship between DII and osteoporosis is unclear.

Moreover, studies evaluating the relationship between bone markers that reflect the process of bone turnover and inflammation related to diet are insufficient. A method for diagnosing osteoporosis using a bone marker has been widely used, but there was no study checking association with DII using both dual-energy X-ray absorptiometry (DXA) and biomarker. Therefore, we examined the association between DII and bone markers in postmenopausal women.

## Material and methods

### Study subjects

This cross-sectional study was conducted among postmenopausal women aged 45–70 years. Participants were recruited from October 2018 to August 2019 at Kyung Hee Medical Center. Postmenopausal women were defined as women after 12 consecutive months without menstruation. Among them, women who did not undergo bilateral oophorectomy or did not take hormones or estrogens became subjects. Subjects with diseases that may affect bone metabolism, undergone hormone replacement therapy or continuously taken osteoporosis-related drugs or dietary supplements within the last 3 months, uncontrolled hypertension patients (Patient with systolic blood pressure ≥160 mmHg despite taking blood pressure medication), body mass index (BMI) of less than 18.5kg/m^2^ or more than 30kg/m^2^, history of spine-related or undergoing surgery on the spine, chronic disease that may affect blood indicators were excluded. Templates were created and posted for recruiting participants and this study was performed for those who voluntarily wished to participate. As a result, a total of 132 participants were recruited, and 4 subjects whose blood parameter data were outside the normal range were excluded. Of the 128 subjects, the final 120 subjects were selected, excluding 8 subjects for whose data such as survey, dietary intake, and blood data were not collected.

This study was approved by the Institutional Review Board (IRB) of the Kyung Hee Medical Center (IRB number: KHUH2018-06-055-003), and all subjects provided written informed consent.

### General characteristics

The general characteristics of the subjects were investigated, including age, age at menopause, number of offspring, alcohol consumption, and smoking status. Alcohol intake was investigated by the frequency of drinking per week, the type of alcohol, and the amount of alcohol consumption. Smoking status was reported using the following categories: Current smoker, Former smoker (status of smoking in the past, but not current), Non-smoker.

### Physical activities

The physical activities were evaluated by a Korean version of the international physical activity questionnaire (IPAQ) Short Form, which indicates the physical activity status and physical activity levels of the subjects in their daily life [30].

We asked subjects to respond to physical activity that lasted at least 10 minutes at a time in the past week, including work, home, transportation, leisure, and exercise. The frequency and the number of vigorous physical activities (carrying heavy things, running, aerobics, riding a bike at high speeds, etc.), moderate physical activity (carrying the light thing, riding a bike at normal speeds, doubles tennis, etc.), walking at least 10 minutes at a time and time spent sitting during the past 7 days were investigated.

Based on IPAQ score conversion guidelines, physical activity time was converted to Metabolic Equivalent of Task (MET-min / week) score [31]. Physical activity time was categorized into walking (3.3 MET score × hours (minutes) × day), moderate (4.0 MET score × hours (minutes) × day), vigorous (8.0 MET score × hours (minutes) × day). MET score of total physical activity was calculated as the sum of walking, moderate and vigorous MET scores.

### Quality of life

The EuroQol- 5 Dimension (EQ-5D) was used to assess the subject’s current health-related quality of life and was proved the validity and reliability by Lee et al. (2009) and Lee et al. (2016) [32, 33]. EQ-5D consists of mobility, self-care, user activity, pain/disability, and anxiety/depression on five categories. It was evaluated on three levels by the current status of ’no problem’, ’some/moderate problem’, ’severe problem’ [32]. Moreover, the quality weight was computed by putting weights on each factor. Quality weight was calculated using the calculation formula suggested by Nam et al. and Lee et al. (2009). The formula according to this model defined the higher the quality of life as the closer the value to 1.

## Dietary assessments and eating habits

A 3-days food record was used to investigate the usual dietary intake of the subjects. Food intake was investigated for 3 days, including 2 days on weekdays and 1 day on weekends. An experienced dietitian told the subjects how to fill out the survey correctly through a 1:1 interview. In order to grasp all kinds of foods and types and the amounts of food intake on the written dietary records, intakes were checked by using additional tools such as food models, measuring cups, and measuring spoons. The daily nutritional intake for each individual was calculated by using a nutrient analysis program (Computer-Aided Nutritional Analysis for Professionals; Can pro, Version 5.0, Korean Nutrition Society). All nutrients were divided into 1000 kcal to exclude differences in energy intake and nutrient-dense [34].

The frequency of meals per day, eating out per week, and overeating per week was investigated to assess the usual eating habits of the subjects.

### Dietary Inflammatory Index (DII)

The DII was developed by Shivappa et al. (2014) to assess the inflammation potential of an individual’s diet [23]. To calculate the inflammatory effect score of food, a review of literature from 1950 to 2010 confirmed the association of inflammatory biomarkers with macronutrients, micronutrients, and phytonutrients. It is a method for scoring from maximum anti-inflammatory to maximum pro-inflammatory, depending on how each food parameter affects the six inflammatory biomarkers (IL-1β, IL-4, IL-6, IL-10, TNF- α, CRP). And then, forty-five food parameters were selected, and it was weighted according to the characteristics of previous studies. Based on the diet data set of 11 countries for food parameters, the global mean and standard deviation of the average meal intake representing the world population were exhibited. The z-score was calculated using the individual diet intake obtained through the dietary survey, the standardized global means, and standard deviation. Of the 45 food parameters, seven food parameters that are not frequently consumed in Korean diets and whose values are unreliable were excluded. The final 38 food parameters were used for the calculation. (S1 Table The 38 food parameters used data from can pro, the national standard food ingredient table of the rural development administration (RDA), Korea food & drug administration (KFDA), and Korea consumer agency. The 38 food parameters included in the calculation are as follows: energy, alcohol, garlic, ginger, onion, green/black tea, pepper, caffeine, flavan-3-ol, flavones, flavonols, flavonones, isoflavones, carbohydrate, cholesterol, saturated fat (SFA), n-3 fatty acids, n-6 fatty acids, polyunsaturated fatty acids (PUFAs), monounsaturated fatty acids (MUFAs), total fat, protein, fiber, magnesium, iron, zinc, selenium, thiamin, riboflavin, niacin, vitamin B_6_, folic acid, vitamin B_12_, vitamin A, β-carotene, vitamin C, vitamin D, vitamin E. Alcohol was calculated as 19 volumes for soju, 4.5 volumes for beer, and 6 volumes for makgeolli (Alcohol consumption (g) = amount of alcohol consumption (mL) × alcohol content (%) × specific gravity of alcohol (0.785)) [35].

The percentile score was calculated to minimize the bias for each nutrient. To achieve a symmetrical distribution with a value of 0, it was calculated by multiplying the percentile score by 2 and subtracting 1 from the value. The ’food parameter’ is yielded by multiplying the estimated ultimate percentile score by the ’overall inflammatory effect score’. The ’food parameter-specific DII score’ of all subjects was summed to yield the final DII score (S1 Fig). The range of scores calculated so far is -8.87 (i.e., strongly anti-inflammatory) to +7.98 (i.e., strongly pro-inflammatory) [23]. In other words, a low DII score means an anti-inflammatory diet, and a high DII score means a pro-inflammatory diet.

### Anthropometric measurements

Body compositions of the subjects were assessed using Bioelectrical Impedance Analysis (BIA; body composition analyzer Inbody 720, Biospace Co, USA). The subjects removed accessories, hats, socks, etc., and wore light clothing. Measurement variables were height, weight, percent body fat (PBF), skeletal muscle mass (SMM). The measured values were recorded by rounding up to 0.1 cm in height and 0.1 kg in weight. Skeletal muscle was assessed using skeletal muscle index-weight (SMIw). SMIw was calculated by appendicular skeletal muscle (ASM) divided by weight and multiplying by 100.

Blood pressure was collected in a comfortable sitting position after the subject remained stable for at least 5 minutes. As measuring, the location of the arm, heart, and machine was put on the same level and measured in relaxed status.

### Biochemical and bone mineral density measurements

Blood samples were drawn from a mid-arm vein after overnight fasting for 8 hours. For blood samples, osteocalcin, alkaline phosphatase (ALP), bone-specific alkaline phosphatase (BSALP), and C-telopeptide of type 1 collagen (CTx) are biochemical markers of bone turnover and were collected to examine the current state of bone. The collected blood was clotted at room temperature for 10 minutes and was centrifuged at 3000 rpm for 10 minutes. The centrifuged blood was analyzed by requesting a specialized institution.

The BMD of lumbar spine L1-L4 and femur total was measured using Dual-energy X-ray absorptiometry (DXA; Lunar iDXA, GE Healthcare Co, Chicago, Illinois, USA). When measuring DXA, the input weight was recorded through the value of a BIA. In this study, BMD, bone mineral content (BMC), T-score, Z-score values were displayed.

According to the World Health Organization (WHO), T-score criteria were classified as normal when it was above -1.0, osteopenia when it was -1.0>T-score>-2.5, and osteoporosis when it was ≤ -2.5.

### Covariates

According to a previous study, variables that could confound the bone health outcomes of postmenopausal women were selected as covariates. The covariates were included age [36], menopause period [37], alcohol consumption [36], smoking status (current smoker, former smoker, non-smoker) [38], physical activity [39], and BMI [36]. Experienced dietitians collected general characteristics by face-to-face interviews, such as age, menopause period, alcohol consumption, and smoking status. Total physical activity time was calculated as METS scores for walking activity, moderate activity, and vigorous activity. BMI was calculated by dividing the weight (kg) by the height (m^2^) using the measured weight and height values.

### Statistical analysis

All data were presented as mean and standard deviation or number and percentages. Chi-square test or Fisher’s exact test was used to examine the relationship between categorical variables, and analysis of variance (ANOVA) was performed to compare the mean of continuous variables. The post hoc analysis was used by the Tukey method. Trend analysis was performed using continuous variable values for each tertile of DII in the general linear model. Linear regression analysis was performed to investigate the relationship between DII score and anthropometric and biochemistry markers. The odds ratios (OR) and 95% confidence interval (CI) were estimated using logistic regression models to investigate the risk of osteoporosis. Statistical analysis was accomplished using SAS version 9.4 (SAS Institute, Cary, NC, USA). All statistical significance was considered p<0.05.

## Results

### General characteristics related to the health-related factors of the subjects

General characteristics of subjects according to tertiles of DII score are described in Table 1. The DII across the tertiles was classified into 3 groups (DII range T1: -5.464 ~ -0.113, T2: -0.060 ~ 1.922, T3: 2.097 ~ 6.772) (p < .0001). The lowest tertile was a less inflammatory potential diet compared to the highest tertiles. The age means of the study subject were 58.1 ± 5.1 years, 57.5 ± 5.0 years, 57.5 ± 5.5 years, respectively. No significant difference was shown in health-related factors (age, age at menopause, first childbirth age, number of offspring, alcohol consumption, smoking status, comorbidity, PA, EQ-5D, and family history of disease).

**Table 1:** General characteristics of the subjects according to the tertiles of DII score.

	T1 (n = 40) (-5.464 ~ -0.113)	T2 (n = 40) (-0.060 ~ 1.922)	T3 (n = 40) (2.097 ~ 6.772)	P-value
Age (y)	58.1±5.1	57.5±5.0	57.5±5.5	0.830
Age at menopause (y)	49.6±3.8	48.3±3.9	49.2±4.0	0.339
First childbirth age (y)	27.3±3.7	27.0±4.7	26.9±3.4	0.881
Number of offspring, n%				
1	5 (27.8)	8 (44.4)	5 (27.8)	0.814 ^e^
2	27 (32.9)	26 (31.7)	29 (35.4)	
> 3	8 (40.0)	6 (30.0)	6 (30.0)	
Alcohol consumption				
Yes	8 (27.5)	10 (27.5)	7 (17.5)	0.849 ^e^
No	28 (70.0)	29 (72.5)	28 (70.0)	
Smoking status				
Current smoker	-	1 (2.5)	2 (5.0)	0.542 ^f^
Former smoker	1 (2.5)	3 (7.5)	1 (2.5)	
Non-smoker	39 (97.5)	36 (90.0)	37 (92.5)	
PA (MET, min/week)	2870.8±3327.8	2381.0±2196.3	2307.8±2887.0	0.630
EQ-5D index score	0.82±0.08	0.82±0.08	0.85±0.06	0.155
Comorbidity^£^	11 (27.5)	11 (27.5)	14 (35.0)	0.700
Family history of disease^₭^	29 (72.5)	32 (80.0)	30 (75.0)	0.727
DII score^¥^	-2.06±1.28 ^a^	0.84±0.57 ^b^	3.60±1.42 ^c^	**< .0001**

All values are presented as mean±standard deviation (SD) or n (%).

^¥^The higher score means pro-inflammatory, and the lower score means anti-inflammatory.

DII: Dietary Inflammatory Index, PA: physical activity

^£^Hypertension, Diabetes Mellitus, hypertriglyceridemia, hyperlipidemia

^₭^Hypertension, Diabetes Mellitus, colorectal cancer, stroke, gastric cancer, renal disease, cervix cancer, esophageal cancer, dementia, hyperlipidemia, prostate cancer, head and neck cancer, lung cancer, liver cancer, asthma, cardiovascular disease, lymphatic carcinoma, breast cancer, gallbladder cancer, tongue cancer, bladder cancer, skin cancer

Statistically significant differences between continuous variables were analyzed using ANOVA, and

a-c post hoc analysis was performed using the Tukey HSD (p<0.05).

^e^ Statistically significant differences in categorical variables were performed using the chi-square test or

f Fisher’s exact test (p<0.05).

MET (min/week): Metabolic equivalents of task

For physical activity, the mean was 2870.8 ± 3327.8 min/week in T1, 2381.0 ± 2196.3 min/week in T2, and 2307.8 ± 2887.0 min/week in T3, respectively, and difference among groups was not observed. For the EQ-5D, there was no statistical significance among groups and also no tendency.

### Dietary factors

Table 2 shows macro- and micronutrient intake for each group according to tertiles of the DII score and eating habits of subjects. The energy intake between each group was 2149.9 ± 479.6 kcal, 1820.1 ± 342.3 kcal, and 1583.5 ± 310.8 kcal, respectively, which was the highest energy intake at T1 (low DII score) compared with T3 (high DII score) significantly (p < .0001). In order to rule out the difference by energy intake, all nutrients were presented divided by per energy 1000 kcal. Compared to T3, the intake of fiber, vitamin A, vitamin E, vitamin K, vitamin C, folic acid, potassium, magnesium, iron, and zinc were higher in T1, and it was statistically significant (p<0.05).

**Table 2:** Dietary factors of the subjects according to the tertiles of DII score^¥^.

	T1 (n = 40) (-5.464 ~ -0.113)			T2 (n = 40) (-0.060 ~ 1.922)			T3 (n = 40) (2.097 ~ 6.772)			P value	P-trend
Total energy (kcal)	2149.9	±	479.6 ^a^	1820.1	±	342.3 ^b^	1583.5	±	310.8 ^c^	**< .0001**	**< .0001**
Carbohydrate (g)	143.6	±	17.6	141.0	±	20.3	143.9	±	21.0	0.833	0.696
Fat (g)	28.1	±	5.9	28.0	±	6.1	28.1	±	6.6	0.728	0.569
n-3 Fatty acids (g)	0.6	±	0.8	0.5	±	0.6	0.4	±	0.5	**0.049**	**0.017**
n-6 Fatty acids (g)	3.4	±	5.1	2.5	±	1.9	2.0	±	1.5	0.254	0.151
Protein (g)	39.2	±	4.6	40.4	±	5.2	37.9	±	5.6	0.760	0.511
Fiber (g)	16.5	±	4.6 ^a^	13.4	±	3.6 ^b^	12.4	±	3.1 ^bc^	**< .0001**	**< .0001**
Vitamin A (μg RAE)	351.1	±	293.0 ^a^	305.9	±	203.3 ^ab^	208.6	±	107.3 ^b^	**0.026**	**0.010**
Vitamin D (μg)	3.9	±	4.3	3.2	±	3.6	2.4	±	3.0	0.132	0.066
Vitamin E (mg)	10.9	±	2.9 ^a^	10.0	±	3.0 ^ab^	9.2	±	2.7 ^b^	**0.001**	**0.000**
Vitamin K (mg)	119.3	±	55.9 ^a^	84.8	±	54.7 ^b^	74.4	±	41.4 ^bc^	**0.002**	**0.001**
Vitamin C (mg)	97.7	±	54.2 ^a^	88.2	±	63.2 ^ab^	67.9	±	44.6 ^b^	**0.046**	**0.009**
Thiamin (mg)	1.1	±	0.2 ^a^	1.0	±	0.2 ^ab^	0.9	±	0.2 ^b^	0.081	**0.028**
Folic acid (μg)	321.8	±	81.9	274.0	±	68.8	257.7	±	67.4	**0.000**	**0.000**
Calcium (mg)	336.5	±	84.5	327.6	±	98.0	287.4	±	112.0	0.261	0.105
Phosphorus (mg)	653.5	±	86.7 ^a^	637.6	±	88.2 ^ab^	601.1	±	106.8 ^b^	0.115	**0.042**
Potassium (mg)	1830.7	±	423.4 ^a^	1583.1	±	308.1 ^bc^	1481.3	±	308.7 ^c^	**< .0001**	**< .0001**
Magnesium (mg)	69.6	±	22.0 ^a^	61.4	±	18.5 ^ab^	50.9	±	17.3 ^b^	**< .0001**	**< .0001**
Iron (mg)	9.6	±	2.4 ^a^	8.7	±	1.9 ^ab^	8.3	±	1.9 ^b^	**0.015**	**0.005**
Zinc (mg)	6.0	±	1.7 ^a^	5.6	±	1.1 ^ab^	5.1	±	0.9 ^b^	**0.028**	**0.008**
**Eating habits**											
Meal frequency / day											
≤ 2 times	4 (10.0)			10 (25.0)			12 (30.0)			0.078	-
3 times	36 (90.0)			30 (75.0)			28 (70.0)				
Eating out frequency/week											
Never	20 (50.0)			13 (32.5)			11 (27.5)			0.068	-
1~2 times	14 (35.0)			19 (47.5)			14 (35.0)				
≥ 3 times	6 (15.0)			8 (20.0)			15 (37.5)				
Overeating frequency/week											
Never or seldom	9 (22.5)			11 (27.5)			11 (27.5)			0.763	-
Sometimes	20 (50.0)			17 (42.5)			14 (35.0)				
Usually	11 (27.5)			12 (30.0)			15 (37.5)				

All values are presented as mean±standard deviation (SD).

^¥^The higher score means pro-inflammatory, and the lower score means anti-inflammatory.

For all nutrients except energy, adjusted DII divided by per energy 1000 kcal was used.

Statistically significant differences between continuous variables were analyzed using ANOVA, and

^a-c^ post hoc analysis was performed using the Tukey HSD (p<0.05).

Statistically significant differences in categorical variables were performed using the Chi-square test (p<0.05).

Trend analysis was performed using continuous variable values, adjusted for age, menopausal period, and BMI, for each tertile of DII in the general linear model.

For the response rate of the Meal frequency, the rate that answered ’Three times a day’ was the highest in the T1 (90%), and the rate that answered ‘≤Two times a day’ was the highest in T3 (30%). However, there was no significant difference among groups. Eating out frequency and overeating frequency were also not statistically significant.

### Anthropometric, biochemistry, and BMD measurements

Anthropometric, biochemistry marker and BMD measurements of subjects by tertiles DII score are described in Table 3. In anthropometric, the average PBF, SMM, SMIw, and handgrip strength showed neither statistically significant nor trends among groups.

**Table 3:** Anthropometric, biochemistry, and bone mineral density measurements of the subjects according to the tertiles of DII score^¥^.

	T1 (n = 36) (-5.464 ~ -0.113)			T2 (n = 40) (-0.060 ~ 1.922)			T3 (n = 34) (2.097 ~ 6.772)			P-value	P-trend
**Anthropometric**											
Height (cm)	155.9	±	4.7	156.7	±	4.1	156.2	±	4.9	0.769	0.785
Weight (kg)	59.3	±	8.7	59.2	±	6.8	60.3	±	7.5	0.785	0.727
PBF (%)	33.4	±	5.6 ^ab^	32.7	±	5.0 ^b^^c^	36.0	±	5.1 ^a^	**0.026**	0.058
SMM (kg)	21.1	±	2.4	21.4	±	2.4	20.7	±	1.8	0.551	0.255
SMIw (%)	26.6	±	2.2	26.9	±	2.0	25.7	±	2.1	0.064	0.080
HGS (kg)	22.5	±	5.7	24.1	±	3.5	23.4	±	3.2	0.253	0.656
**Biochemistry marker**											
Osteocalcin (ng/mL)	21.02	±	7.58	18.57	±	5.88	18.57	±	5.36	0.171	0.088
ALP (IU/L)	66.14	±	12.97 ^a^	60.00	±	11.38 ^b^	56.18	±	13.34 ^b^^c^	**0.005**	**0.001**
BSALP (ug/L)	16.63	±	4.82 ^a^	15.11	±	3.61 ^ab^	13.34	±	3.63 ^b^	**0.004**	**0.001**
CTx (pg/mL)	0.45	±	0.18	0.45	±	0.16	0.39	±	0.13	0.198	0.100
Ca (mg/dL)	9.19	±	0.25	9.13	±	0.32	9.17	±	0.24	0.670	0.691
P (mg/dL)	3.98	±	0.27	3.87	±	0.40	3.83	±	0.29	0.150	**0.042**
Vitamin D (ng/mL)	19.53	±	9.07	20.10	±	7.02	17.65	±	5.59	0.345	0.304
PTH (pg/mL)	36.82	±	10.12	37.34	±	8.69	38.38	±	9.24	0.776	0.413
**BMD**											
**Lumbar spine 1–4**											
BMD (g/cm^2^)	0.95	±	0.15	1.01	±	0.17	0.97	±	0.12	0.216	0.510
BMC (g)	52.01	±	10.54	55.57	±	10.44	54.21	±	7.35	0.275	0.308
T-score	-1.63	±	1.25	-1.13	±	1.39	-1.45	±	1.04	0.215	0.511
Z-score	-0.71	±	1.14	-0.24	±	1.36	-0.57	±	0.92	0.193	0.601
**Total femur**											
BMD (g/cm^2^)	0.90	±	0.11	0.93	±	0.11	0.93	±	0.11	0.577	0.390
BMC (g)	26.86	±	3.98	27.38	±	3.56	27.28	±	3.52	0.808	0.555
T-score	-0.58	±	0.96	-0.36	±	0.95	-0.40	±	0.95	0.583	0.392
Z-score	-0.26	±	0.87	-0.06	±	0.95	-0.11	±	0.72	0.589	0.479

All values are presented as mean±standard deviation (SD).

^¥^The higher score means pro-inflammatory, and the lower score means anti-inflammatory.

PBF: percent body fat, SMM: skeletal muscle mass, SMIw (skeletal muscle index-weight): (appendicular skeletal muscle ÷ weight)×100, HGS: handgrip strength, ALP: alkaline phosphatase, BSALP: bone-specific alkaline phosphatase, CTx: C-telopeptide of type 1 collagen, Ca: calcium, P: phosphorus, PTH: parathyroid hormone, BMD: bone mineral density, BMC: bone mineral content

Indices of Ca, P, serum Vitamin D, and PTH excluded those subject to the abnormal range.

Statistically significant differences between continuous variables were analyzed using ANOVA, and

^a,b^ post hoc analysis was performed using the Tukey HSD (p<0.05).

Trend analysis was performed using continuous variable values, adjusted for age, menopausal period, and physical activity, for each tertile of DII in the general linear model.

The bone-related markers (Ca, P, 25-OH vitamin D, parathyroid hormone (PTH)) involved in homeostasis were excluded beyond the normal range. The average of the bone formation markers ALP was 66.14 ± 12.97 IU/L in T1, 60.00 ± 11.38 IU/L in T2, and 56.18 ± 13.34 IU/L in T3, respectively and the average of BSALP was 16.63 ± 4.82 ug/L in T1, 15.11 ± 3.61 ug/L in T2, and 13.34 ± 3.63 ug/L in T3, respectively. There were statistically significant differences among groups, and those tend to decrease from T1 to T3 (p <0.05).

The BMD of the lumbar spine from L1 to L4, BMD of the total femur neck showed no statistical differences and no tendency among the groups. The BMC, T-score, and Z-score of the lumbar spine also showed no significant difference and no trend among groups. No significant and tendency were also shown in the total femur.

### Association between DII score and anthropometric, biomarker and BMD

Table 4 shows the association between the DII score and the anthropometric, biochemistry marker, and BMD. The association between DII score and anthropometric, the unadjusted crude value was not statistically significant. As age, menopause period, alcohol consumption, smoking status, BMI, and PA were adjusted in multivariate, and with higher DII score, PBF increased (β = 0.168, p = 0.012) and SMIw was decreased (β = -0.329, p = 0.037).

**Table 4:** Association between DII score and anthropometric, biomarker and BMD.

DII score						
	Crude			Multivariate ^a^		
	β	SE	P	β	SE	P
**Anthropometric**						
PBF (%)	0.074	0.044	0.090	0.168	0.065	**0.012**
SMM (kg)	-0.097	0.110	0.380	-0.182	0.134	0.176
SMIw (%)	-0.153	0.111	0.170	-0.329	0.156	**0.037**
**Biochemistry marker**						
Osteocalcin (ng/mL)	-0.034	0.037	0.373	-0.047	0.040	0.245
ALP (IU/L)	-0.055	0.018	**0.002**	-0.057	0.018	**0.002**
BSALP (ug/L)	-0.152	0.055	**0.007**	-0.167	0.056	**0.004**
CTx (pg/mL)	-1.552	1.490	0.300	-2.038	1.572	0.198
Ca (mg/dL)	-0.344	0.871	0.694	-0.348	0.911	0.703
P (mg/dL)	-1.277	0.719	0.079	-1.799	0.797	**0.026**
Vitamin D (ng/mL)	-0.026	0.032	0.419	-0.027	0.036	0.446
PTH (pg/mL)	-0.002	0.026	0.933	0.004	0.027	0.870
**BMD**						
**Lumbar spine 1–4**						
BMD (g/cm^2^)	-0.204	1.291	0.875	-0.334	1.399	0.812
BMC (g)	0.001	0.020	0.973	-0.003	0.022	0.877
T-score	-0.024	0.155	0.878	-0.039	0.168	0.816
Z-score	-0.056	0.165	0.733	-0.030	0.171	0.859
**Total femur**						
BMD (g/cm^2^)	0.117	1.694	0.945	-0.088	1.976	0.964
BMC (g)	-0.027	0.053	0.603	-0.036	0.061	0.563
T-score	0.012	0.203	0.952	-0.013	0.237	0.957
Z-score	-0.026	0.226	0.911	0.009	0.240	0.969

PBF: percent body fat, SMM: skeletal muscle mass, SMIw (skeletal muscle index-weight): (appendicular skeletal muscle ÷ weight)×100, ALP: Alkaline phosphatase, BSALP: bone-specific alkaline phosphatase, CTx: C-telopeptide, Ca: calcium, P: phosphorus, PTH: parathyroid hormone, BMD: bone mineral density, BMC: bone mineral content

^a^ Trend analysis was performed using continuous variable values, adjusted for age, menopausal period, smoking status, alcohol consumption, BMI, and physical activity, for each tertile of DII in the general linear model.

For crude biochemistry markers, linear regression analysis revealed a decrease in ALP and BSALP as the DII score increased (β = -0.055, p = 0.002 in ALP, β = -0.152, p = 0.007 in BSALP). In the multivariate model, ALP, BSALP, and P were decreased as the DII score increased (β = -0.057, p = 0.002 in ALP, β = -0.167, p = 0.004 in BSALP, β = -1.799, p = 0.026 in P). No association was observed between the DII score and BMD.

### Risk of osteopenia and osteoporosis according to DII score

Table 5 shows OR and respective 95% CI risks of osteopenia, osteoporosis, and osteopenia+osteoporosis. A logistic regression analysis was performed to investigate the relationship of DII score to the risk of osteopenia and osteoporosis in postmenopausal women. The risk of osteopenia increased as the DII score increased (OR 2.06, 95% CI 1.099–3.914). In model 1, the risk of osteopenia increased as DII score increased when the age and menopausal period were adjusted (OR 2.12, 95% CI 1.126–4.041), and in model 2, the risk of osteopenia increased as well, when BMI and physical activity was additionally adjusted (OR 2.13, 95% CI 1.119–4.093). As the DII score increased, the risk of osteoporosis was decreased when the confounder variable was adjusted. However, there was no relationship between DII and osteopenia+osteoporosis.

**Table 5:** Binary logistic regression for the relationship between DII score and risk of osteopenia and osteoporosis.

	DII score (n = 120)		
Variable	OR	95% CI	P-value
**Osteopenia**	2.06	(1.099, 3.914)	**0.024**
Mode1 1	2.12	(1.126, 4.041)	**0.021**
Mode1 2	2.13	(1.119, 4.093)	**0.022**
**Osteoporosis**	0.47	(0.223, 0.963)	**0.044**
Mode1 1	0.42	(0.191, 0.907)	**0.031**
Mode1 2	0.41	(0.182, 0.896)	**0.029**
**Osteopenia+osteoporosis**	1.21	(0.600, 2.432)	0.595
Mode1 1	1.27	(0.604, 2.650)	0.523
Mode1 2	1.26	(0.589, 2.712)	0.540

OR = odds ratio; CI = confidence interval

Trend analysis was performed using continuous variable values for each tertile of DII in the general linear model.

Model 1 was adjusted for age, menopause period, smoking status, alcohol consumption

Model 2 was adjusted for model 1, BMI, and Physical Activity

## Discussion

This study is a cross-sectional study that confirmed the association between DII and bone markers in postmenopausal women in Korea. Interestingly, we found that people with low DII scores prefer a diet rich in dietary fiber, vitamins, and minerals. Additionally, after adjusting age, menopause period, and BMI, we observed a significant negative relationship between low DII score and body fat and a positive association with muscle mass; the lower the DII score, the higher the bone biomarkers after adjusting confound factors. There was no association between DII and BMD, but it was associated with an increased risk of osteopenia as DII increased.

Intake of vitamins and minerals lowers inflammatory biomarkers and regulates inflammatory status [13]. Our study is consistent with previous studies that inflammation is closely related to diet [40]. A cohort study showed that there was a high intake of energy, PUFA, n-3 fatty acid, vitamins, and minerals in the anti-inflammatory diet group [41]. Similarly, the anti-inflammatory group had a high intake of total energy, vitamins, and minerals in 40–49 years adults [24]. Several studies have suggested that foods with high cholesterol and processed foods are closely related to pro-inflammatory foods, but this could not be confirmed in our study [16–18].

DII is associated with body weight and anthropometric indicators [42]. Furthermore, DII had a clear association between abdominal obesity in women compared to men [42]. Adherence to a Mediterranean diet, a diet high in whole grains, fruits, vegetables, dairy products, and olive oil, is associated with inflammatory markers [43]. In a study that identified the relationship between the Mediterranean diet and muscle mass, muscle mass was improved in the group with a high Mediterranean diet score (high intake of food known as the Mediterranean diet). The decreasing body fat and the increased muscle mass in the low DII score are consistent with our study, but more research is needed to establish the relationship between DII and muscle mass in postmenopausal women.

There has been no previous study between low DII and bone markers, but the increase in bone markers in the low DII score group can explain the following reasons. Previous studies reported that osteocalcin increased when an oil-rich Mediterranean diet was highly consumed [44]. In addition, the role of vitamin K and vitamin D is important for osteocalcin, which is a bone formation marker, to synthesize and function. In the synthesis process of osteocalcin, vitamin K regulates the carboxylate process according to the intake, and vitamin D directly induces the synthesis of osteocalcin. Dietary intake of vitamin K and vitamin D in the anti-inflammatory group was high, estimated to affect bone formation marker concentration [45]. This is consistent with our findings that vitamin D and vitamin K intake were high in groups with low DII score. Although not significant, osteocalcin tended to be lower as the DII score was higher. These studies may support the rise of bone turnover markers in the anti-inflammatory group. In this regard, we confirmed that ALP and BSALP were low in high DII scores. Studies on the possibility of inflammation of diet and bone markers are currently insufficient, and it is known that pro-inflammatory cytokines are involved in decreasing BMD, but the relationship with bone markers is unclear.

Several recent studies have commenced taking an interest in the relationship between DII and BMD. Another study showed that BMD decreased as DII score increased, and the risk of osteoporosis increased in the femoral region [29]. Similar results were observed among Iranian women, and the pro-inflammatory diet increases the risk of decreasing BMD [28]. In addition, in the study of the elderly population, pro-inflammatory foods were confirmed to increase the risk of fracture [46]. However, in the Brazilian osteoporosis study, females had a higher DII score than males, but there was no association between DII score and fracture risk of vulnerability [47]. In our study, there was no association between DII and BMD. However, as DII increased, it was associated with an increased risk of osteopenia, but osteoporosis did not. The lower risk of osteoporosis is expected to be due to excluding serious osteoporosis patients from this study.

The inflammatory responses and bone remodeling are complex interactions mediated by various hormones, cytokines, and minerals [48]. After menopause, cytokines that are involved in bone resorption for various reasons are activated [3, 4]. This process is fast for someone and slow for someone. Foods generally known to help bone health and foods known as anti-inflammatory foods are similar. A balanced diet is more important than trying to find foods related to reducing inflammation or increasing BMD. Various nutrients and foods are involved in reducing inflammation, but nutrients and foods interact with each other [40]. It is also important to take an overall diet rather than a single nutrient or food [20]. Hence, it can be dangerous to judge by just one factor because various lifestyle factors are closely related to each other [49].

There are several limitations to this study. First, the causal relationship between DII and bone markers could not be confirmed as a cross-sectional design. The socioeconomic data that could affect the dietary intake was also not considered because it was not collected. Second, only 38 of the 45 parameters were available to calculate the DII score. The food parameters such as eugenol, saffron, and thyme are those that do not use are not frequently consumed in this region. Previous studies showed that the validity of DII did not change, even though 28 out of 45 were used [50]. Third, inflammatory biomarkers could not be identified. Nonetheless, studies on the relationship between DII and inflammatory biomarkers have been verified in previous studies. DII alone has been able to identify the possibility of inflammation of the bone marker and the diet [51–53]. Finally, the number of subjects was small, which might lead to a weak statistical inference.

Despite these limitations, the strength of this study is the first study conducted in Korea to evaluate the association between the inflammatory potential of diets and bone markers. It is also worth noting that DXA and bone markers were measured together to identify BMD in postmenopausal women.

## Conclusion

Our findings suggest that a low DII score is positively associated with low body fat mass, high muscle mass, elevated bone markers, and low risk of osteopenia. However, it is still unclear how bone markers are affected by dietary intake. Therefore, further studies are needed to determine the association between the possibility of inflammation of the diet and bone markers and lifestyle factors.

## Supporting information

S1 Fig Scoring process of DII. (TIF)

S1 Table Items included in the components of DII. (TIF)

S1 Dataset (TXT)
