# Supplementary figures and images for: Association between the dietary inflammatory index and bone markers in postmenopausal women
Source: PLoS One. 2022 Mar 17;17(3):e0265630. doi: 10.1371/journal.pone.0265630 (PMC8929634; doi:10.1371/journal.pone.0265630)

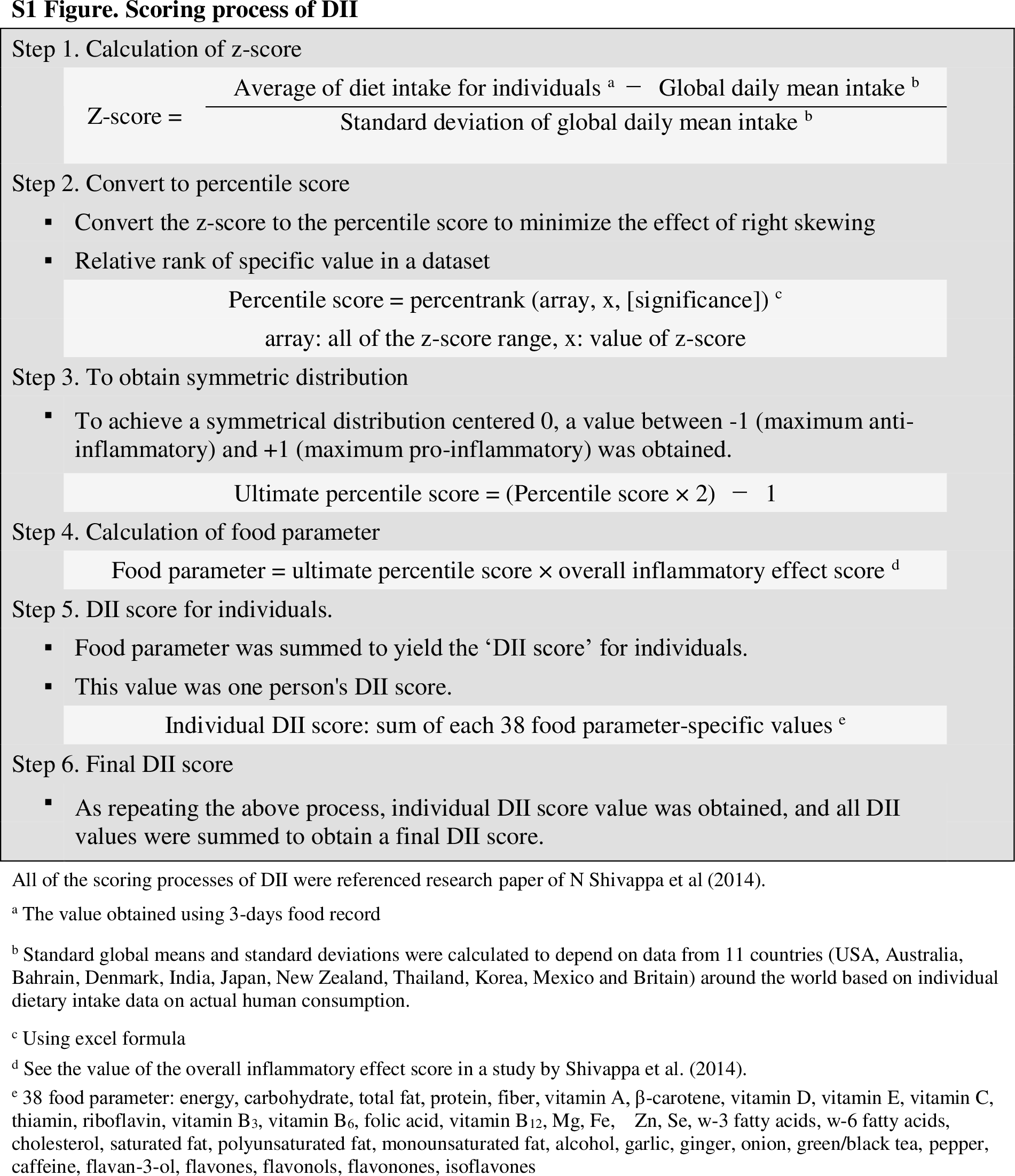

Supplement: S1 Fig — (TIF) [file pone.0265630.s001.tif]

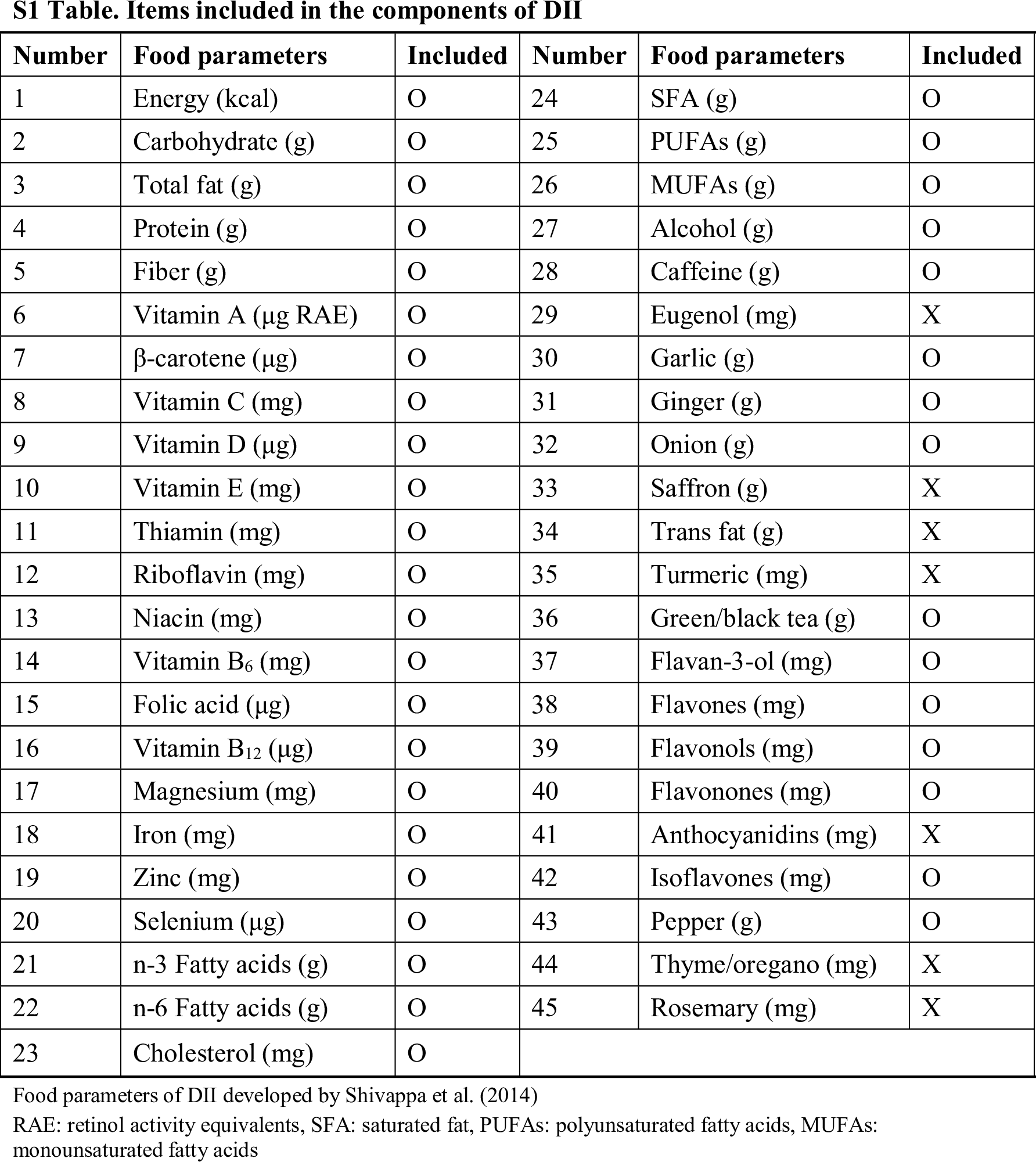

Supplement: S1 Table — (TIF) [file pone.0265630.s002.tif]
